# The Edinburgh variant of a talar body fracture: a case report

**DOI:** 10.1186/1749-799X-5-92

**Published:** 2010-12-09

**Authors:** Nicholas D Clement, Sally-Ann Phillips, Leela C Biant

**Affiliations:** 1 Department of Orthopaedics and Trauma, The Royal Infirmary of Edinburgh, Little France, Edinburgh EH16 4SA, UK

## Abstract

We describe a novel closed pantalar dislocation with an associated sagittal medial talar body and medial malleolus fractures. Closed reduction was attempted unsuccessfully. Open reduction was performed, revealing a disrupted talonavicular joint with instability of the calcaneocuboid joint. This configuration required stabilisation with an external fixator. There were no signs of avascular necrosis, or arthrosis at 15 months follow but is currently using a stick to mobilise.

## Introduction

Talar fractures account for 0.3% of all fractures, with an incidence of 3.2 per 100,000 and are predominantly a male injury (82:18) [1]. Talar body fractures occur in only 7% to 38% of all talar fractures [2-10]. Sneppen et al [11] classified talar body fractures into five distinct groups: compression (talocrural joint), shearing (coronal or sagittal), posterior tubercle, lateral tubercle and crush fractures. The Orthopaedic Trauma Association [12] and Delee [13] have since further classified these fractures, but no classification to date recognises a pantalar dislocation associated with a talar body facture.

We describe a previously unclassified closed pantalar dislocation with an associated sagittal medial talar body and medial malleolus fractures.

## Case report

**Figure 1:**
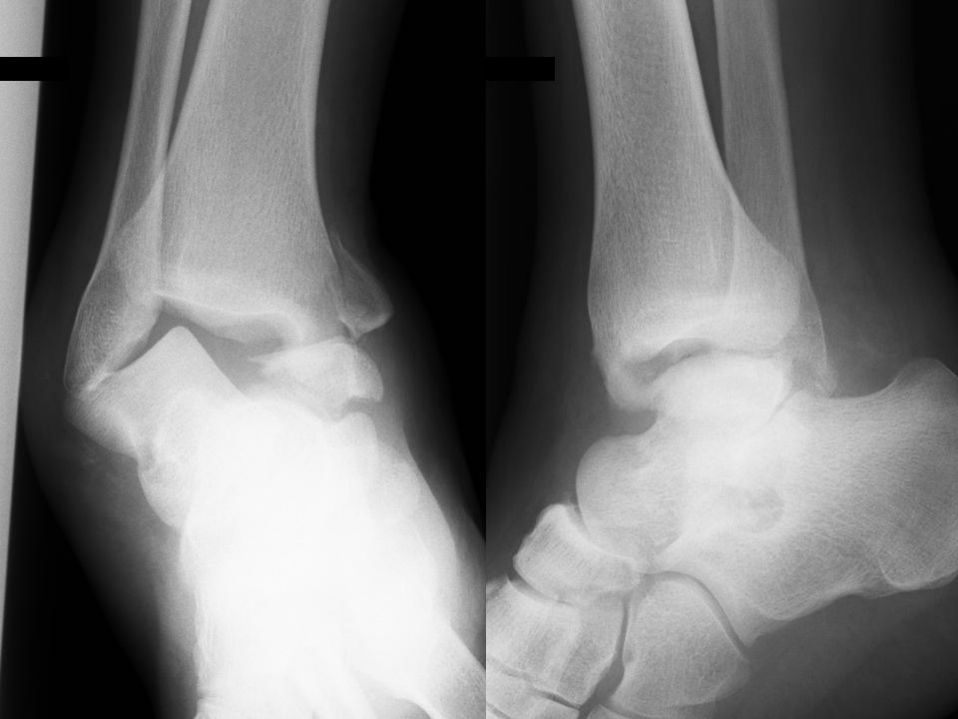
**Anterio-posterior and lateral radiograph at time of presentation**.

**Figure 2:**
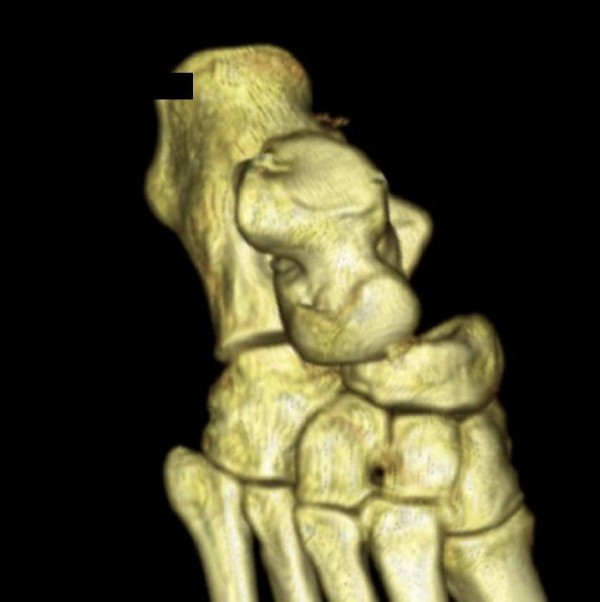
**Three dimensional computerised tomography reconstruction scan pre-operatively with the tibia and fibular removed**.

A 32 year old postman fell whilst walking in a forest, sustaining a hyper plantar flexion and external rotation injury to his right ankle. He presented to the Accident and Emergency department with a grossly swollen and deformed right ankle. The skin was intact, with a minor abrasion over the lateral malleolus. There was no neurovascular deficit. Radiographs demonstrated a fracture of the talar body and the medial malleolus with dislocation of the talus (Figure 1). After two failed attempts at closed reduction under sedation in the emergency department we abandon further attempts to avoid additional soft tissue damage and any further insult to the residual blood supply to the talar body. An urgent computerised tomography scan was obtained with subsequent three dimensional reconstruction (Figure 2).

**Figure 3:**
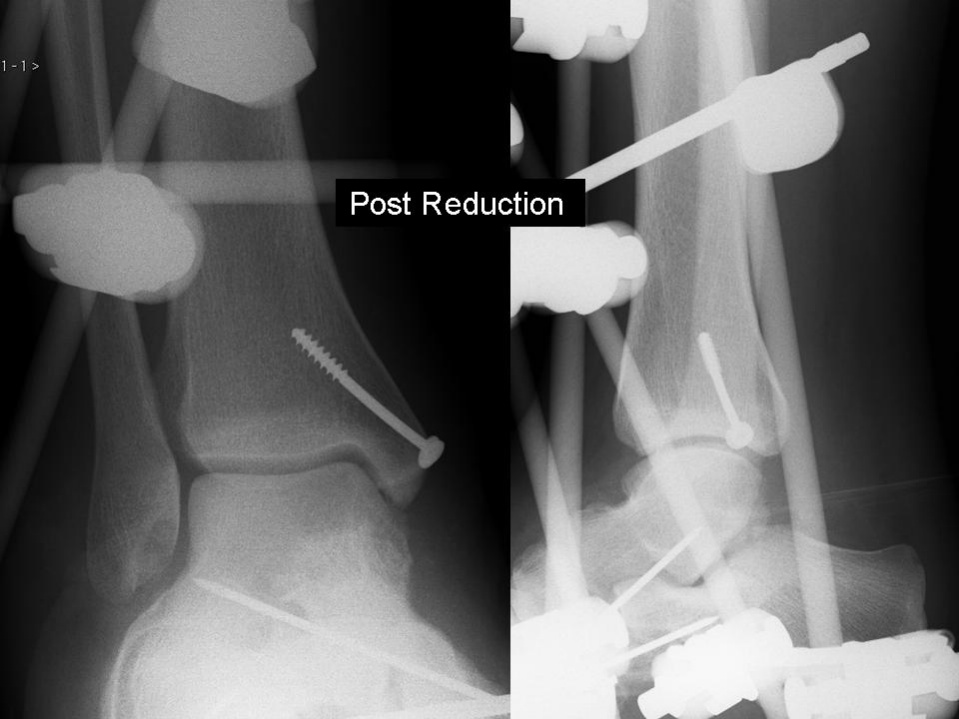
**Anterio-posterior and lateral radiograph post reduction**.

**Figure 4:**
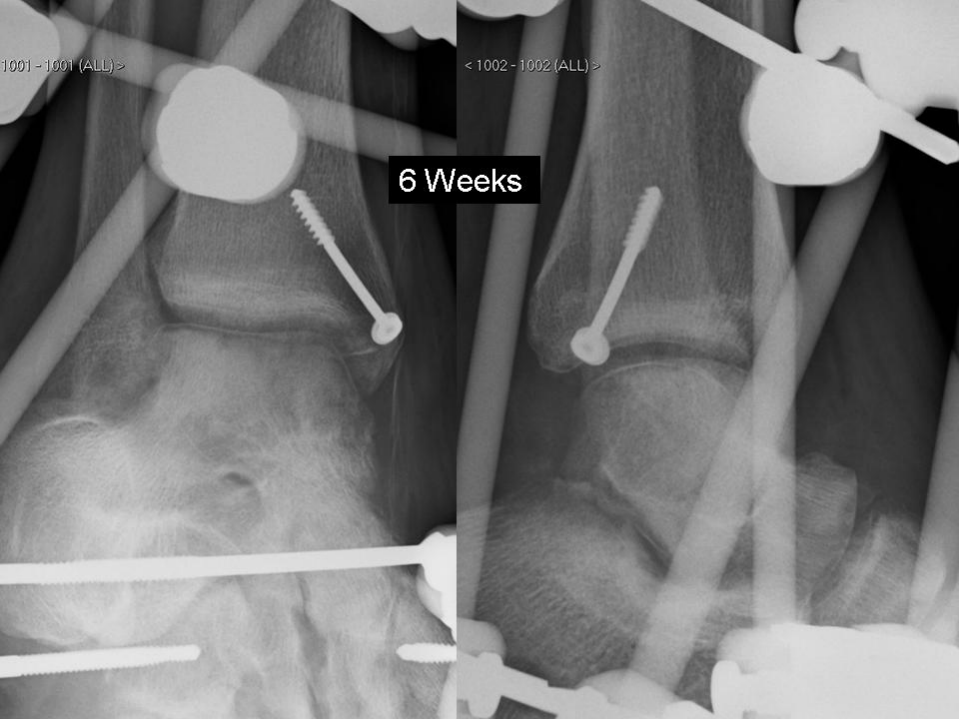
**Anterio-posterior and lateral radiograph at 6 weeks**.

**Figure 5:**
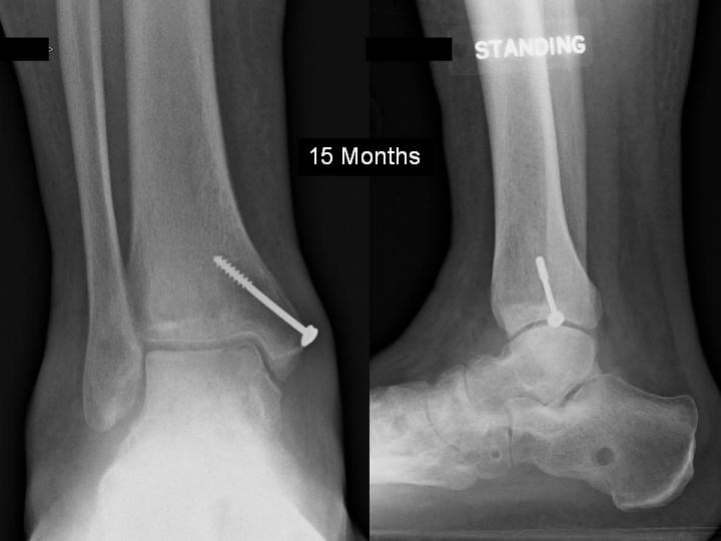
**Anterio-posterior and lateral radiograph at 15 months**.

Six hours after presentation open reduction was performed primarily through an anteromedial approach, a medial malleolar osteotomy was not necessary as this was already fractured giving adequate access, as described by Rammelt and Zwipp [14]. The posterior medial fragment was comminuted and fixation was no possible, the fragments were excised. The talonavicular joint was not reducible and a further anterolateral approach was made to enable reduction. The calcaneocuboid joint was unstable, so Kirschner (K) wires were used to hold the reduction. Despite this the talonavicular joint remained unstable and a bridging external fixator was used to hold the reduction (Figure 3). The medial malleolus was fixed with a single screw. He remained non-weight bearing for 6 weeks where upon the frame and K-wires were removed. Radiographs at 6 weeks (Figure 4) demonstrated Hawkins sign, with no signs of avascular necrosis or arthrosis at 15 months follow up (Figure 5). The range of movement continues to improve, the current range is: plantar flexion 20 degrees, dorsiflexion 10 degrees, inversion 20 degrees, and eversion 10 degrees, with full power (5/5 MRC scale) in all planes. He currently has minimal pain (4/10 on the visual analogue scale), tending to be after prolonged standing/walking. He has not yet returned to full employment and still uses a stick to mobilise.

## Discussion

We describe a novel variant of a talar body fracture: closed pantalar dislocation with an associated sagittal medial talar body and medial malleolus fractures. To date no classification has described this fracture pattern. Hafez et al. [15] described a similar fracture pattern. They report a closed coronal fracture through the body of the talus with pantalar dislocation; the talus had "rotated 90 degrees laterally" in the transverse plane. Whereas, we observed a sagittal fracture and a pantalar dislocation with rotation in a coronal plane (Figure 2).

A unique aspect of this case was the observed instability of the calcaneocuboid joint, which is widened in Figure 2. We feel this was torn open superiorly with the hyper plantar flexion, allowing the talar head to dislocate. After reduction the talonavicular joint remained unstable, due to plantar flexion opening the unstable calcaneocuboid joint and required stabilisation with an external fixator.

Our case demonstrated Hawkins sign at 6 weeks post injury, which is a sign of remodelling and is highly predictive of revitalisation of the talar body: radiolucent zone at in the subcortcal bone of the talar dome (Figure 4) [14]. Avascular necrosis is a complication that would be expected following such an injury pattern [16]. However, injuries associated with a medial malleolus fracture, as we have described are less likely to develop avascular necrosis. This is due to preservation of the deltoid ligament and the associated deltoid branch of the posterior tibial artery supplying the talar body [17,18].

The prognosis of talar fractures/dislocations is related to the severity of the injury, length of time before relocation and early fixation. The infection rate varies depending on definition, from 3.1% deep infection rate to 6.2% if superficial infections are also included [19]. The majority infections occur after an open fracture which carries a worse prognosis [20]. The risk of avascular necrosis of the talar body is related to the type of fracture, with non-displaced talar body fractures being associated with a 5% to 44% risk, whereas displaced talar body fractures the risk is about 50% [16], which is further increase if the injury is open [21,22]. Post-traumatic arthrosis varies from 16 to 100% after talar body fractures [21,23]. Malunion can produce significant alteration in load across the ankle and subtalar joints and result in arthrosis [21]. Anatomic and stable reduction of talar body fractures is of paramount importance for obtaining a reasonable functional outcome [21]. There is no apparent correlation between talar body fracture classification and outcome, which maybe explained by the low incidence and variation of such injuries [14]. Approximately 80% patients will have good to excellent clinical results after early internal fixation [23]. The reported case, according to the aforementioned criteria, should have a good prognosis as it was closed and underwent immediate operative reduction with early signs of revascularisation.

This case presents a new variant of talar body fracture, with a new rotatory element and a disruption of the calcaneocuboid joint. Urgent open reduction should be employed with adequate imaging to plan the approach and potential fixation of the fracture.

## Consent

Written informed consent was obtained from the patients for publication of this case report and any accompanying images. A copy of the written consent is available for review by the Editor-in-Chief of this journal.

## Competing interests

The authors declare that they have no competing interests.

## Authors' contributions

LCB is the surgeon in charge of the patient and helped with editing the report. SAP and NDC (corresponding author) wrote the original report and performed a literature review. All authors have read and approved the final manuscript
